# Advancements in Artificial Intelligence in Noninvasive Cardiac Imaging: A Comprehensive Review

**DOI:** 10.1002/clc.70087

**Published:** 2025-01-27

**Authors:** Oluwaremilekun Zeth Tolu‐Akinnawo, Francis Ezekwueme, Olukunle Omolayo, Sasha Batheja, Toluwalase Awoyemi

**Affiliations:** ^1^ Department of Internal Medicine Meharry Medical College Nashville Tennessee USA; ^2^ Department of Internal Medicine University of Pittsburgh Medical Center McKeesport Pennsylvania USA; ^3^ Department of Internal Medicine Lugansk State Medical University Lugansk Ukraine; ^4^ Department of Internal Medicine Government Medical College Patiala Punjab India; ^5^ Department of Internal Medicine Feinberg School of Medicine, Northwestern University Chicago Illinois USA

**Keywords:** AI (artificial intelligence), cardiac imaging techniques, health care reforms, precision medicine

## Abstract

**Background:**

Technological advancements in artificial intelligence (AI) are redefining cardiac imaging by providing advanced tools for analyzing complex health data. AI is increasingly applied across various imaging modalities, including echocardiography, magnetic resonance imaging (MRI), computed tomography (CT), and nuclear imaging, to enhance diagnostic workflows and improve patient outcomes.

**Hypothesis:**

Integrating AI into cardiac imaging enhances image quality, accelerates processing times, and improves diagnostic accuracy, enabling timely and personalized interventions that lead to better health outcomes.

**Methods:**

A comprehensive literature review was conducted to examine the impact of machine learning and deep learning algorithms on diagnostic accuracy, the detection of subtle patterns and anomalies, and key challenges such as data quality, patient safety, and regulatory barriers.

**Results:**

Findings indicate that AI integration in cardiac imaging enhances image quality, reduces processing times, and improves diagnostic precision, contributing to better clinical decision‐making. Emerging machine learning techniques demonstrate the ability to identify subtle cardiac abnormalities that traditional methods may overlook. However, significant challenges persist, including data standardization, regulatory compliance, and patient safety concerns.

**Conclusions:**

AI holds transformative potential in cardiac imaging, significantly advancing diagnosis and patient outcomes. Overcoming barriers to implementation will require ongoing collaboration among clinicians, researchers, and regulatory bodies. Further research is essential to ensure the safe, ethical, and effective integration of AI in cardiology, supporting its broader application to improve cardiovascular health.

## 1 Introduction and Background

**Figure 1:**
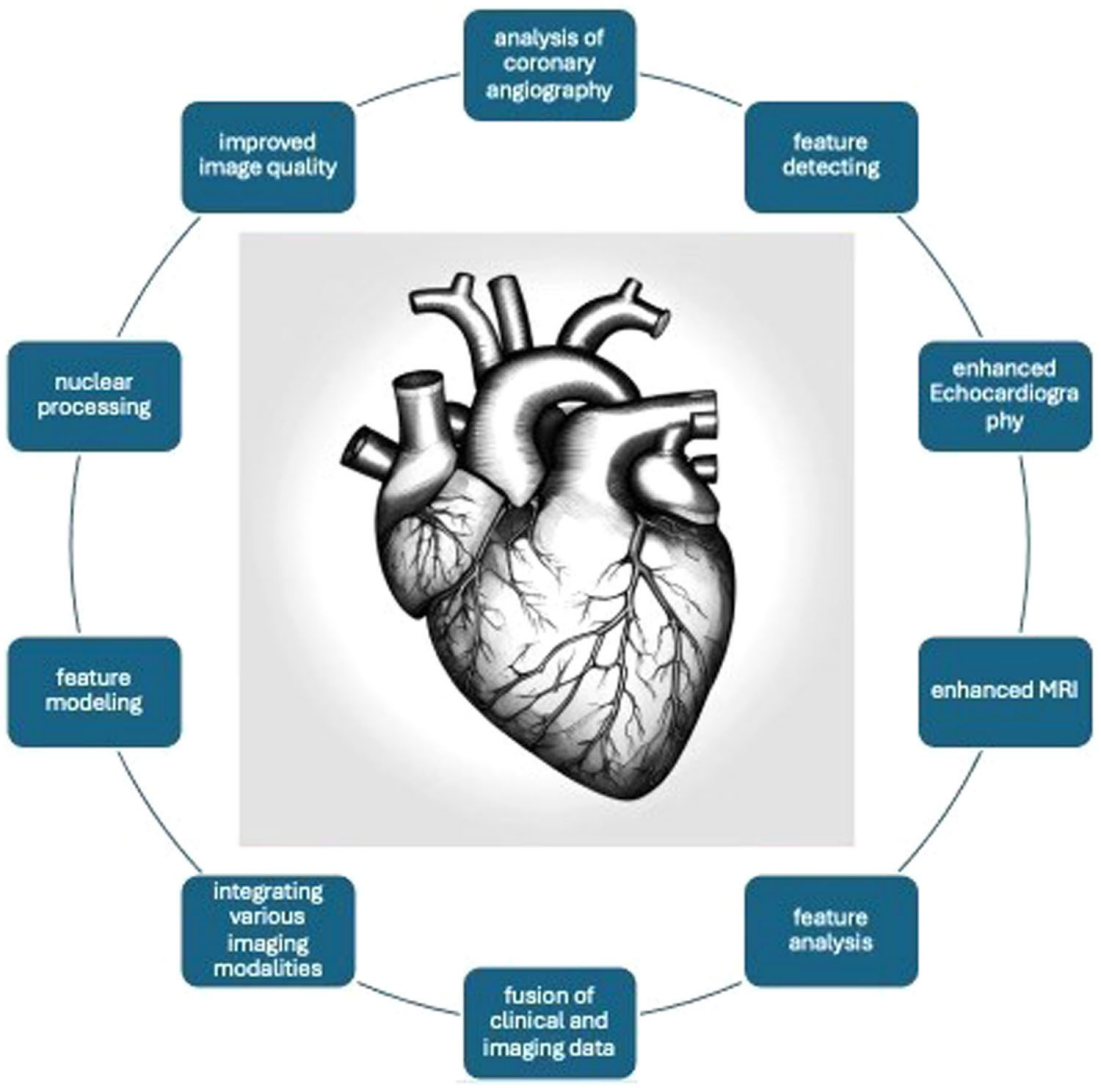
The figure illustrates AI application in noninvasive cardiac imaging. *Source:* Francis Ezekwueme: one of the authors.

In recent years, the healthcare industry has witnessed an unprecedented surge in the application of artificial intelligence (AI), revolutionizing diagnostic precision, treatment strategies, and administrative operations [1]. By leveraging sophisticated algorithms and extensive data sets, AI has immense potential to transform healthcare delivery, enhance patient outcomes, and ultimately save lives. Studies have demonstrated that AI algorithms can match or even surpass human capabilities in various healthcare tasks, including analyzing medical images, correlating symptoms with biomarkers, and predicting diseases using electronic medical records [2]. In cardiovascular medicine, AI has significantly advanced cardiac imaging, leading to breakthroughs in diagnostic accuracy and treatment planning [3]. Advances in cardiac imaging technology, ranging from traditional echocardiography to advanced modalities like cardiac magnetic resonance imaging (MRI) and computed tomography (CT) angiography, have empowered clinicians to visualize cardiac anatomy and function with unprecedented detail and accuracy [4]. These advancements enable early detection of heart conditions and play a pivotal role in guiding therapeutic interventions and monitoring treatment as illustrated in Figure 1.

AI optimization enhances image quality, expedites processing times, and improves diagnostic precision [1] while addressing critical challenges such as data integrity, patient safety, and regulatory compliance. Machine learning (ML) and deep learning (DL) algorithms play a central role in identifying intricate patterns and anomalies that conventional methods may overlook. These algorithms optimize diagnostic accuracy by analyzing vast data sets and aid in personalized medicine, ultimately improving patient outcomes. The profound impact of AI on personalized medicine and patient outcomes is thoroughly reviewed in this article. Highlighting the evolving landscape of AI‐driven cardiac diagnosis underscores the need for ongoing research and collaborative efforts to effectively harness AI's potential in cardiac care. These efforts drive healthcare innovation to new heights, promising a future where AI is central to optimizing cardiac imaging and improving patient care [3]. This has facilitated a more integrated and holistic approach to healthcare, providing a multidisciplinary pathway to the cardiology community where teams can collaborate effectively to provide the best care for patients and improve healthcare efficiency.

This review aims to explore the integration of AI across various cardiac imaging modalities, including echocardiography, MRI, CT, and nuclear imaging, the challenges of implementation, and its potential benefits in improving cardiac health outcomes.

## 2 Review

### 2.1 Selection Process

The criteria for articles selection were established using factors such as publication date, relevance to topic, the study design, and geographic scope as a guide for selection. We searched data databases such as PubMed, Google Scholar, and Web of Science by using keywords: “Artificial Intelligence,” “Cardiac Imaging” to extract relevant articles. After selecting the initial articles, we checked for duplicates, and then the titles/abstracts/full texts were extensively reviewed to identify articles that met our inclusion criteria which include full‐text articles that discussed AI and cardiac imaging, healthcare technological advances, and cardiac imaging. We excluded articles which did not meet these criteria. After selecting the articles for review, we then examined the reference lists of our selected articles to identify additional relevant sources that could be helpful to our comprehensive review of the topic. The quality of the final selected articles was assessed by evaluating the study design, methodology, sample size, and author credibility. Findings from these selected articles were helpful in formulating an extensive review on this topic “AI‐Powered Cardiac Imaging.”

### 2.2 Advanced Techniques in Cardiac Imaging

Cardiac imaging encompasses a spectrum of techniques, from conventional methods to cutting‐edge innovations. Echocardiography, MRI, CT, and nuclear imaging are pivotal modalities in contemporary cardiac imaging, each offering unique advantages in visualizing cardiac anatomy, assessing function, and diagnosing pathological changes with remarkable precision [5]. Recent advancements in hardware and software have made coronary artery calcium (CAC) scoring CT, coronary CT angiography (CCTA), and cardiac MRI (CMR) more accessible in primary care settings. Faster postprocessing and manipulation of large data sets enable clinicians to address critical clinical questions efficiently and accurately [5].

Despite technological progress, traditional methods like echocardiography, nuclear imaging, and invasive angiography have notable limitations [6]. Echocardiography, while noninvasive, is operator‐dependent and limited by the acoustic window, impacting the field of view [7]. While providing functional information, nuclear imaging techniques such as positron emission tomography (PET) and CT can be costly and inaccessible in certain healthcare settings. Although considered the gold standard, invasive angiography poses risks to patient safety and discomfort due to its invasive nature [8, 9].

Innovations in cardiac imaging, particularly with advanced techniques like cardiac MRI and CT angiography, offer superior spatial resolution and anatomical details. However, challenges remain regarding cost, availability, and interpretation expertise. AI presents a promising solution to these challenges by enhancing diagnostic accuracy. AI algorithms complement existing cardiac imaging technologies by improving image quality, expediting processing times, and detecting intricate patterns and anomalies that may evade human interpretation [10].

### 2.3 AI‐Powered Tools in Echocardiography

ML, a subfield of AI, endows computers with the ability to learn from data without explicit programming through techniques such as supervised and unsupervised learning [11]. In supervised learning, the model is trained on a labeled data set, which means that each training example is paired with an output label. The algorithm learns to map inputs to the desired output, making it effective for tasks like classification and regression [11]. On the other hand, unsupervised learning involves training on data without labels, allowing the model to identify patterns and relationships within the data, which is helpful for clustering and association tasks. ML employs various statistical methods, including regression, which predicts a continuous outcome variable based on one or more predictor variables, and decision trees, which split data into subsets based on the value of input features to make decisions or predictions [12]. More sophisticated techniques, such as neural networks, consist of interconnected layers of nodes (neurons) that process data in a manner inspired by the human brain [13]. These networks can learn to recognize intricate patterns in data through a process of training and adjustment of weights. DL is an advanced subset of ML that utilizes specialized neural network architectures known as deep neural networks [14]. These architectures consist of multiple layers that automatically extract significant features from raw data, thereby eliminating the need for manual feature engineering [14]. This capability makes DL particularly well‐suited for complex tasks like image recognition and natural language processing, where the high dimensionality and variability of the data make manual feature extraction impractical.

In cardiology, ML and DL algorithms significantly enhance the processes of myocardial segmentation and quantification, reducing operator dependency and variability [15]. Traditionally, the manual segmentation of cardiac structures, including the left ventricle (LV) and right ventricle (RV), for quantification has been labor intensive and subject to interobserver variability [16]. This process requires expert knowledge and considerable time, making it prone to human error and inconsistencies. mEcho‐AI applications involve multiple sequential processing steps, including view labeling, quality control, segmentation of cardiac structures, measurement of echocardiographic parameters, and disease diagnosis [17]. With manual reading, boundary identification remains a significant concern, thus, limiting accuracy. The creation of the AI algorithms that mimick what an experienced human eye and brain can do, instead of endocardial bordering tracing [18]. The AI‐echo systems can also help identify standard apical views, perform timing of cardiac events, trace myocardium, and perform motion estimation at a faster rate than traditional reading [18]. These applications streamline the analysis of echocardiographic data, reduce operator workload, enhance accessibility to cardiac imaging, and provide real‐time guidance for clinicians thus improving mortality prediction than manual measurement [18]. By automating these steps, Echo‐AI ensures that the analysis is consistent and accurate, regardless of the operator's experience level [19].

AI‐driven solutions also facilitate the early detection of myocardial abnormalities and valvular diseases by offering quantified measurements that improve diagnostic accuracy [18]. For instance, these tools can detect subtle changes in cardiac structures and function that might be missed by the human eye, providing an additional layer of scrutiny that enhances overall diagnostic capabilities. Furthermore, AI algorithms can analyze large data sets quickly, providing immediate feedback and insights that support clinical decision‐making. Not just that, AL technology can be applied to classify and recognize intracardiac masses such as left atrial thrombus cardiac tumors and vegetations [18]. This could be potentially missed by traditional echocardiography interpretation, especially in situations with complex and diverse anatomical structures of the left atrial appendages and limited range of motion of the transesophageal echocardiogram (TEE) probe leading to devastating outcomes [18]. A recent study by Sun et al. demonstrated a superior outcome in the diagnosis of the left atrial thrombosis in patients with atrial fibrillation through the use of artificial neural network model versus the traditional TEE [18].

The primary rationale behind the use of AI in echocardiography lies in its ability to identify diseases, automatically analyze features from images as well as data beyond the human perception [20]. During the routine echocardiography, a lot of vital diagnostic information can be underutilized which could be due to the extensive data generated that is hard to interpret by human experts within a short period of time. Advances in AI‐driven integration can help identify the true values of these “missed” findings, while helping to analyze faster within a shorter period compared to the human expert [20]. This has been particularly helping in cardiovascular disease (CVD) processes such as valvular heart diseases, coronary artery disease (CAD), hypertrophic cardiomyopathy, cardiac malignancies, and amyloidosis diseases.

In a recent study involving about 2000 patients with aortic stenosis, Al integrated echocardiographic measurements were responsible for the improved classification of disease severity and high‐risk groups (through higher aortic valve calcium scores, larger gadolinium enhancement, higher biomarker levels) which helped optimize the timing of aortic valve replacements [21]. Not just that, another recent study highlighted the impact of DL algorithms in classifying echocardiographic views, detecting valvular heart diseases, and quantify disease severity with high accuracy (AOC > 0.88 for all left heart valve disease) compared to the human experts [22]. Recent advances have also shown potential benefit of the ML in obtaining high classification accuracy in the identification of patients with severe CAD which have heightened its integration in the analysis of stress echocardiograms in providing automated classifications, improve accuracy and provider reader confidence [23]. Also, recent advances have shown a potential benefit of AI integration in differentiating two similar disease pathologies, for example, differentiating acute myocardial infarction from Takotsubo syndrome [24]. Also, AI models have shown encouraging signs in providing a predicting of left ventricular recovery after coronary syndromes which could help identify patients at risk and in need of closer clinical monitoring and potentially decreasing post coronary syndrome mortality and morbidity rates [25]. Further potential benefits of AI model in echocardiography including in intracardiac masses, cardiomyopathies, etiology determination of increased left ventricular wall thickness, although have shown potential benefits, however, need additional investigations.

### 2.4 AI in Cardiac MRI

Cardiac MRI is a noninvasive diagnostic tool renowned for identifying a wide range of heart conditions with high precision. Utilizing high‐resolution imaging and employing magnets and radio waves, cardiac MRI avoids the risks associated with radiation‐based imaging techniques, making it a preferred choice over echocardiography and cardiac CT in many clinical scenarios.

#### 2.4.1 Clinical Applications and Effectiveness

Cardiac MRI excels in diagnosing numerous cardiac conditions due to its superior imaging capabilities:

*Congenital Defects:* Cardiac MRI provides detailed anatomical images crucial for diagnosing congenital heart anomalies, offering insights that can guide surgical planning and intervention [20].

*Valvular Defects:* The high‐resolution images help assess valvular morphology and function, aiding in diagnosing and managing valvular heart diseases [26].

*Heart Failure:* Cardiac MRI is invaluable in evaluating heart failure because it provides comprehensive data on myocardial structure, function, and viability [26].

*Tumors:* Cardiac MRI can differentiate between various types of cardiac masses, aiding in the diagnosis of tumors and guiding therapeutic decisions [26].

*Vascular Disorders and Abnormal Growth:* The imaging modality's ability to visualize blood vessels and detect abnormalities such as aneurysms and vascular malformations makes it essential to manage vascular disorders [26].

#### 2.4.2 Limitations and Challenges

Despite its advanced technological capabilities, cardiac MRI faces several limitations that can impact its diagnostic performance and increase costs:

*Motion Artifacts:* Patient movement can compromise the quality of cardiac MRI images, necessitating precise coordination between the imaging process and the patient's breathing cycle [27].

*Lengthy Imaging Times:* The process of acquiring high‐resolution images can be time‐consuming, often requiring patients to remain still for extended periods, which can be challenging for some individuals [27].

*Noise and Claustrophobia:* The MRI machines can emit significant noise during scanning, and the confined space can induce claustrophobia in some patients, affecting their comfort and ability to undergo the procedure [28].

*Adverse Reactions to Contrast Agents:* While rare, some patients may experience adverse reactions to the gadolinium‐based contrast agents used in MRI [28].

*Metallic Implants:* Metallic implants can interfere with the magnetic fields used in MRI, complicating the imaging process, and sometimes precluding its use altogether [28].

#### 2.4.3 The Transformative Role of AI in Cardiac MRI

Cardiac MRI is widely regarded as the gold standard for assessing cardiac function due to its ability to provide detailed images of the heart's structure and function, offering unparalleled insights in the diagnostic workup of suspected CVD. However, despite its high diagnostic value, cardiac MRI is also one of the most challenging imaging modalities to interpret, largely due to the complexity of cardiac motion [29]. The heart's continuous movement during the cardiac cycle, combined with variations in anatomical structures, makes accurate and consistent interpretation a demanding task, often requiring significant expertise and experience.

In a recent study by Wang et al., a pioneering investigation was conducted into the use of AI for the interpretation of cardiac MRI. The study involved a large cohort of 8066 patients diagnosed with various forms of CVD and 1653 individuals with no known cardiac conditions, serving as controls [30]. The AI system developed in this study was designed to screen and diagnose cardiac anomalies by analyzing the MRI data.

The findings of Wang et al. are particularly noteworthy, as they demonstrated that the AI system could detect cardiac anomalies with a level of accuracy comparable to, and in some cases superior to, that of experienced cardiologists [30]. This study not only highlights the potential of AI to enhance diagnostic accuracy but also underscores the efficiency gains that can be achieved by integrating AI into the diagnostic process. The AI was able to rapidly process and interpret large volumes of imaging data, which is crucial in clinical settings where timely diagnosis can significantly impact patient outcomes and potentially limiting the burden on cardiologists.

Moreover, the study's findings suggest that AI could play a pivotal role in standardizing the interpretation of cardiac MRI, reducing variability between different operators, and ensuring consistent diagnostic quality across various healthcare settings [30]. This could be particularly beneficial in resource‐limited settings, where access to highly experienced cardiologists may be limited. The AI system's ability to assist less experienced clinicians in making accurate diagnoses could help bridge disparities in cardiovascular care.

The implications of Wang et al.'s study are far‐reaching, pointing to a future where AI could become an integral part of the diagnostic workflow in cardiology. By enhancing the accuracy and efficiency of cardiac MRI interpretation, AI has the potential to improve patient outcomes, streamline clinical workflows, and ultimately contribute to better management of CVDs [30]. This study is a significant step forward in the application of AI in medical imaging and sets the stage for further research and development in this rapidly evolving field.

The accurate quantification of biophysical parameters also is a significant benefit of AI in MRI. Also, a potential benefit is auto segmentation of cardiac structures and pathology in an improved accuracy compared to the traditional methods [29].

Integrating AI into cardiac MRI has the potential to revolutionize the imaging workflow, enhancing its efficiency, accuracy, and diagnostic capabilities:

*Enhanced Image Generation:* AI algorithms, particularly those involving ML and DL, can process large data sets to optimize image generation. This improvement results in clearer and more detailed images, facilitating more accurate diagnoses [31].

*Detection of Subtle Conditions:* AI‐driven systems can identify subtle changes and patterns in cardiac structures that might be overlooked by human observers, thus improving the early detection and management of heart conditions [31].

*Reduced Imaging Time:* AI can streamline the imaging process, reducing the time required to acquire high‐quality images. This reduction in imaging time enhances patient comfort and increases the throughput of MRI machines, making the process more efficient [31].

*Cost Efficiency:* By optimizing various aspects of the imaging workflow, AI can help reduce operational costs, making cardiac MRI more accessible and cost‐effective [31].

#### 2.4.4 Challenges in AI Integration

While the benefits of AI in cardiac MRI are substantial, several challenges must be addressed to fully realize its potential:

*High Computational Costs:* Developing and implementing sophisticated AI algorithms require significant computational resources, which can be a barrier, particularly in resource‐limited settings [32].

*Data Privacy and Security Risks:* The reliance on large data sets for training AI models raises concerns about data privacy and security. Protecting patient data is paramount to maintaining trust and compliance with regulatory standards [32].

*Potential Biases:* AI systems are only as good as the data on which they are trained. If the training data sets are biased, the resulting AI models can produce skewed outcomes, potentially exacerbating health disparities [32].

*Resistance to Adoption:* Healthcare professionals and the public may be resistant to adopting AI technologies due to concerns about reliability, interpretability, and the potential for job displacement. Addressing these concerns through education and demonstrating the tangible benefits of AI is crucial [32].

### 2.5 AI in Cardiac CT

CT coronary angiography is a noninvasive, painless diagnostic imaging tool that utilizes low levels of X‐ray radiation to capture detailed images of cardiac and coronary structures and functions. Cardiac CT is widely valued for its cost‐effectiveness and rapid image production, which is crucial for timely diagnosis and treatment planning. However, the interpretation of these images traditionally relies heavily on the expertise of clinicians, which can introduce variability and potential delays in diagnosis.

AI, particularly through ML algorithms such as convolutional neural networks (CNNs), can revolutionize cardiac CT by enhancing image construction and providing more conclusive diagnoses. These AI‐driven technologies can significantly speed up the imaging process, enabling radiologists to achieve a quicker workflow and output. This acceleration in workflow is critical as it allows clinicians to initiate early patient interventions, potentially improving outcomes [33].

In addition, computed tomography‐derived fractional flow reserve (CT‐FFR) using on‐site ML has shown significant potential in identifying both the presence of CAD and vessel‐specific ischemia. A recent study involving 1216 patients with stable CAD and intermediate stenosis (30%–90%) on CT angiography compared the effectiveness of an on‐site CT‐FFR care pathway using ML to standard care across 6 Chinese medical centers [34]. The study found that 421 out of 608 patients (69.2%) in the CT‐FFR group underwent invasive coronary angiography, compared to 483 out of 608 patients (79.4%) in the standard care group [34].

Importantly, the CT‐FFR group had a significantly lower proportion of patients with nonobstructive CAD or obstructive CAD not undergoing invasive coronary angiography compared to the standard care group (28.3% [119/421] vs. 46.2% [223/483]; *p* < 0.001) [34]. Additionally, more patients in the CT‐FFR group underwent revascularization (49.7% [302/608]) compared to the standard care group (42.8% [260/608]; *p* = 0.02), indicating greater efficiency and effectiveness. Major adverse events were similar between both groups. Notably, the CT‐FFR group also incurred lower costs due to the reduced need for invasive angiography [34].

#### 2.5.1 Benefits of AI in Cardiac CT

*Faster Image Construction and Diagnosis:* AI algorithms, especially CNNs, can process large data sets rapidly, constructing images faster than traditional methods. This speeds up the diagnostic process and allows for real‐time adjustments and interpretations, facilitating quicker clinical decisions [33].

*Reduced Radiation Exposure:* One of the significant advantages of AI in cardiac CT is its ability to produce high‐quality images with reduced radiation exposure. AI algorithms can optimize imaging parameters and enhance image clarity, ensuring accurate diagnostics with minimal radiation doses compared to conventional methods [35].

*Minimized Motion Artifacts:* AI technologies can effectively reduce motion artifacts, which are common in cardiac imaging due to the constant movement of the heart. By improving image stabilization and clarity, AI ensures more precise and reliable diagnostic outcomes [11].

*Automation of Imaging Processes:* AI can automate several critical processes in cardiac CT imaging, including segmentation, risk stratification, and assessing CAC levels. These automated processes enhance efficiency and ensure consistency and accuracy in diagnostics [36].

*Advanced 3D Reconstruction and Visualization:* AI enables capturing more images along multiple planes, facilitating advanced 3D reconstruction and visualization of cardiac structures. This comprehensive view aids in better diagnosis and treatment planning, providing clinicians with detailed insights into cardiac conditions [17].

#### 2.5.2 Challenges and Considerations

Despite the numerous benefits, the integration of AI into cardiac CT also presents several challenges that need to be addressed to realize its full potential:

*Variability in Diagnostic Standards:* There is significant variability in diagnostic standards across different institutions, which can hinder the consistent application of AI technologies. Establishing a unified gold standard for AI‐assisted cardiac CT imaging is crucial for widespread adoption and reliability [37].

*Data Sharing and Standardization:* The success of AI in cardiac CT heavily relies on data sharing and the standardization of practices. Collaborative efforts are needed to create extensive, diverse data sets that can train robust AI algorithms and ensure their generalizability across various clinical settings [37].

*Quality Assurance and Validation:* While AI can automate many processes, human oversight remains essential. Radiologists must proofread and validate AI‐generated results to maintain high standards of care and ensure that AI technologies complement rather than replace clinical expertise [36].

*Ethical and Regulatory Compliance:* The deployment of AI in healthcare must comply with ethical guidelines and regulatory standards to ensure patient safety and data privacy. Ongoing collaboration between AI developers, clinicians, and regulatory bodies is necessary to navigate these complex landscapes effectively [37].

### 2.6 AI in Nuclear Cardiology

Nuclear cardiology is a specialized field that uses radionuclides for diagnostic imaging of myocardial perfusion through techniques such as PET and single photon emission computed tomography (SPECT). Myocardial perfusion imaging (MPI) stress testing is crucial in evaluating coronary blood flow and identifying obstructions, making it an essential tool for risk stratification in cardiac patients.

#### 2.6.1 Enhancing Imaging Workflow With AI

The integration of AI throughout the nuclear cardiology workflow offers significant enhancements in various aspects of imaging and diagnosis:

*Improved Emission Detection:* AI algorithms can significantly enhance the detection of radionuclide emissions. By optimizing the detection process, AI can improve the quality of images obtained from PET and SPECT scans, making it easier to accurately identify and analyze myocardial perfusion patterns. This enhancement is critical for obtaining clear and detailed images that are essential for accurate diagnosis [38].

*Reduced Radionuclide Usage:* One of the major benefits of AI in nuclear cardiology is its potential to reduce the amount of radionuclides required for imaging. AI can optimize the imaging process to ensure that high‐quality images are obtained with lower doses of radionuclides, thereby reducing patients' exposure to radioactive materials. This reduction in dosage not only enhances patient safety but also makes the imaging process more efficient and cost‐effective [38].

*Accurate Image Registration:* PET and SPECT imaging often need to be correlated with CT imaging to improve diagnostic accuracy. AI can facilitate the precise registration of these images, ensuring that they align correctly and provide comprehensive insights into the patient's cardiac condition. This accurate registration is crucial for combining anatomical and functional data to offer a complete picture of myocardial health [38].

*Early Identification of At‐Risk Patients:* AI's ability to detect subtle changes in myocardial perfusion is instrumental in identifying patients at risk of cardiac events early. By analyzing imaging data in detail, AI can distinguish true positive diagnoses from false positives, reducing the potential for diagnostic errors. This early identification helps implement timely interventions, significantly improving patient outcomes [39, 40].

*Enhanced Predictability of Outcomes:* AI contributes to the predictability of patient outcomes by providing detailed and accurate assessments of myocardial perfusion. These insights enable healthcare providers to make informed decisions about patient management and treatment plans, enhancing the overall effectiveness of cardiac care [39, 40].

#### 2.6.2 Challenges and Considerations

Despite the promising advancements, the integration of AI in nuclear cardiology is not without challenges:

*Data Quality and Standardization:* Ensuring the quality and consistency of data used to train AI algorithms is paramount. Variations in imaging protocols, equipment, and patient demographics can introduce biases and affect the performance of AI models. Standardizing data collection and processing methods across institutions is essential to develop reliable and generalizable AI tools [30].

*Computational Resources:* AI algorithms, particularly those involving DL, require substantial computational resources for training and deployment. The need for high‐performance computing infrastructure can be a barrier, especially in resource‐limited settings. Investing in robust computational platforms and cloud‐based solutions can help overcome this challenge [39].

*Ethical and Regulatory Compliance:* The deployment of AI in nuclear cardiology must adhere to ethical guidelines and regulatory standards to ensure patient safety and data privacy. Navigating the complex regulatory landscape requires ongoing collaboration between AI developers, clinicians, and regulatory bodies to establish clear guidelines and best practices [40].

*Acceptance by Healthcare Professionals:* Resistance to adopting AI technologies among healthcare professionals can hinder the integration process. It is crucial to provide adequate training and demonstrate the tangible benefits of AI in improving diagnostic accuracy and workflow efficiency to gain acceptance and trust from medical practitioners [39].

### 2.7 AI for Remote Disease Monitoring and Telemedicine

Telemedicine involves delivering healthcare services using advanced technology, addressing critical factors such as accessibility and distance. This approach became particularly prominent during the coronavirus‐19 (COVID‐19) pandemic when isolation measures were necessary to curb the spread of the virus. Although initially driven by the pandemic, telemedicine has continued to grow and is now increasingly encouraged in various healthcare settings. AI, particularly DL, has been pivotal in enhancing telemedicine due to its autonomous learning capabilities and adaptability. Unlike traditional computer programs, AI can process and analyze complex data and images more efficiently, leading to significant advancements in healthcare technology [41]. Telemonitoring, which involves the use of digital platforms to monitor patients remotely, has seen substantial improvements with the integration of AI. These AI platforms enable continuous outpatient monitoring, thereby enhancing disease management and patient care [42].

AI has notably improved mortality outcomes by enabling earlier and more accurate predictions of critical health events. For instance, ML approaches have been able to predict cardiac arrest 24 h earlier than traditional modified warning scores in critically ill patients in the Intensive Care Unit (ICU) or Emergency Room (ER). This early prediction capability is crucial for timely interventions and improving patient survival rates [43]. During the COVID‐19 pandemic, the FDA emergency approved AI‐driven wearable and mobile EEG technologies for monitoring QT intervals in patients taking medications like azithromycin or hydroxychloroquine. This approval underscores AI's critical role in ensuring patient safety and enhancing clinical monitoring capabilities [43]. Through telehealth and remote monitoring technologies, AI has sustained healthcare coverage and stability for patients with physical limitations. These innovations have ensured continuous and effective healthcare delivery, even for those unable to visit healthcare facilities in person.

## 3 Challenges of Integrating AI Tools Into Existing Clinical Practices

Integrating AI tools into existing clinical practices involves overcoming a variety of challenges, ranging from technological barriers to cultural resistance. Addressing these challenges is crucial to ensure that AI can effectively and ethically enhance patient care and clinical outcomes.

### 3.1 Technological Challenges

#### 3.1.1 Data Integration

Clinical environments often involve disparate systems and data formats. Integrating AI tools requires seamless integration with electronic health records (EHRs), imaging databases, and other clinical systems. This can be complicated by the lack of standardization in data formats and interoperability issues.

#### 3.1.2 Infrastructure Requirements

AI systems typically require substantial computational power and advanced hardware, including high‐performance servers and GPUs. Upgrading existing infrastructure to accommodate these requirements can be expensive and time‐consuming. This is particularly difficult in low‐resource settings and may further bridge health disparity.

#### 3.1.3 Data Quality and Consistency

AI algorithms rely on high‐quality, consistent data to function effectively. Inconsistent data entry, missing information, and varied data sources can degrade AI tools' performance. Ensuring data quality through rigorous validation and standardization processes is essential.

#### 3.1.4 Real‐Time Processing

Many clinical decisions need to be made in real time, requiring AI systems to process and analyze data quickly and accurately. Ensuring that AI tools can deliver real‐time insights without compromising performance is a significant challenge. Also, the paucity of real‐time support makes AI adoption by clinicians particularly challenging.

### 3.2 Ethical and Regulatory Challenges

There are four primary ethical concerns with AI use in healthcare: data privacy and security, informed consent for the use of personal data and information, algorithm fairness and biases, and safety and transparency [44].

#### 3.2.1 Data Privacy and Security

In the world of data‐driven healthcare systems, privacy remains a critical challenge especially given the utilization of ML and DL systems in making predictions using user's data [45]. Patients must be able to trust providers to protect their private information such as age, sex, and health data [46]. Protecting patient data is critical to the full integration of AI into cardiac care. AI‐based applications have direct impact on patient's confidentiality [46] and finding the ideal balance between data access restrictions and use of patient's data is crucial. AI systems must comply with stringent data privacy regulations such as HIPAA in the United States or General Data Protection Regulation (GDPR) in Europe. A recent study by Priyanshu et al. highlighted how instructing AI technologies such as ChatGPT to adhere with HIPAA and GDPR and ensure the output generated are compliant and observing the extent of personally identifiable information (PII) omission from responses [45]. Ensuring data security involves implementing robust encryption, secure data storage, and stringent access controls is also critical.

#### 3.2.2 Regulatory Approval

Due to the potential challenges and ethical concerns involved, AI tools in healthcare must undergo rigorous evaluation and approval by regulatory bodies like the FDA or EMA. This process can be lengthy and complex, involving extensive testing to demonstrate safety, efficacy, and compliance with regulatory standards.

#### 3.2.3 Bias and Fairness

Transparency and bias continue to be a barrier in its adoption. This is because AI algorithms can inherit biases from training data, leading to discriminatory outcomes. Less‐represented population data may also lead to false outcomes. Ensuring fairness requires ongoing monitoring and adjustment of AI models to mitigate bias and ensure equitable treatment across diverse patient populations.

#### 3.2.4 Transparency and Explainability

Transparency is also another significant ethical concern in AI integration into healthcare due to lack of decision‐making processes. Striking a balance between accuracy and explainability is critical especially in situations that involve high‐risk decision‐making [45]. AI decision‐making processes must be transparent and explainable. Explainability of AI is the ability of AI‐driven system to be able to provide an understanding of how it arrived at certain prediction or conclusion [45]. In healthcare, explainability involves two steps. One is understanding how the system reaches its conclusion while the second step involves explaining the training process that enables the system to learn from examples and produce outputs [45]. Legally informed consent, certification, approval and liability are crucial aspects of explainability of medical devices and there are currently various explainable AI (XAI) applications currently being studied [45]. There are also several specific algorithms used to enhance interpretation in AI‐integrated medical imaging [45]. These are steps currently ongoing to improve the transparency and explainability of AI models. It is imperative clinicians understand how AI tools arrive at their conclusions in order to trust and effectively use them in clinical practice. Developing XAI models builds clinician confidence and ensures ethical use.

### 3.3 Cultural and Organizational Challenges

#### 3.3.1 Resistance to Change

Healthcare professionals may be resistant to adopting new technologies, particularly if they perceive AI as a threat to their expertise or autonomy. Overcoming this resistance requires demonstrating the value of AI in enhancing clinical decision‐making and patient outcomes, while also ensuring safety in the care of the patients.

#### 3.3.2 Training and Education

Integrating AI tools necessitates comprehensive training for clinicians and support staff. This includes understanding how to use AI systems, interpreting their outputs, and integrating them into clinical workflows. Continuous education and support are essential for successful adoption.

#### 3.3.3 Workflow Integration

AI tools must seamlessly integrate into existing clinical workflows without causing disruptions. This involves designing user‐friendly interfaces, ensuring compatibility with current systems, and minimizing additional workload for clinicians.

#### 3.3.4 Collaboration and Communication

Effective integration of AI tools requires collaboration between clinicians, data scientists, and IT professionals. Clear communication and collaboration are essential to addressing technical issues, aligning expectations, and ensuring that AI tools meet clinical needs.

### 3.4 Financial and Economic Challenges

#### 3.4.1 High Implementation Costs

Cost remains an important challenge to the integration of AI in cardiac imaging. This is because AI‐powered tools in cardiac imaging often require significant upfront investments in technology, infrastructure, and training. The initial investment includes but not limited to software, hardware, training, and ongoing maintenance costs. Ensuring a return on investment involves demonstrating long‐term cost savings through improved efficiency and patient outcomes.

#### 3.4.2 Cost of Upgrading Infrastructure

In order to ensure accurate diagnosis and outcomes, it is important to ensure existing clinical infrastructure is continuously upgraded to support AI tools, including high‐performance computing resources and data storage solutions. This can be a major financial burden, particularly for smaller healthcare providers. Unfortunately, patients from these populations have less access to the resources being enjoyed by their richer/higher counterparts, further bridging health disparities.

#### 3.4.3 Cost Savings

Over time, AI can potentially save costs by improving diagnostic accuracy, reducing the need for repeat imaging, and minimizing operator workload. Also, enhanced efficiency and reduced manual labor can result in lower operational costs.

#### 3.4.4 Accessibility in Resource‐Limited Settings

The access to AI technologies may be limited in resource‐constrained settings due to high costs. Ensuring equitable access to AI tools across different healthcare settings requires developing cost‐effective solutions and promoting international collaboration to share resources and expertise.

High implementation costs may pose a barrier to the adoption of AI‐powered tools in resource‐limited settings. Strategies to improve accessibility include developing cost‐effective AI solutions, leveraging cloud‐based platforms to reduce local infrastructure costs, and promoting international collaboration to share resources and expertise.

#### 3.4.5 Long‐Term Economic Impact

The long‐term economic benefits of AI in cardiac imaging include improved patient outcomes, reduced hospital stays, and lower overall healthcare costs due to more accurate and timely diagnoses. Wider adoption of AI technology may lead to economies of scale, reducing costs and increasing accessibility over time. Table 1 highlights the different modalities of AI‐cardiac imaging modalities, their benefits, and challenges.

**Table 1:** Summary of Al‐cardiac imaging modalities.

Aspect	Description	Benefits	Challenges	Economic aspects
Echocardiography	This involves the use of ML and DL for myocardial segmentation, view labeling, quality control, and disease diagnosis	− Reduces operator dependency and variability − Enhances diagnostic accuracy and real‐life guidance − Automates segmentation and qualification. − Improves accessibility to cardiac imaging.	− There is potential for coding errors and misclassification biases. − Limited ability to control all potential confounders. − Lack of detailed clinical information may limit interpretation.	− High initial implementation cost remains a primary challenge. − Also potential cost savings from reduced manual labor and improved efficacy. − Accessibility challenges in resource‐limited settings due to high costs.
Cardiac MRI	Al integration enhances image generation, improves image quality, and reduces imaging time and costs.	− Provides high‐resolution imaging. − Effective for diagnosing a wide range of heart conditions. − Reduced imaging time and costs. − Improves image reconstruction using historical data.	− High computation cost. − Data privacy and security risks − Resistance to adoption by healthcare professionals and the public	− High setup and maintenance costs for Al technology. − Long‐term cost savings from improved efficiency and reduced need for repeat imaging. − High costs may limit use in low‐resource settings.
Cardiac CT	Al, especially CNNs, for faster image construction, conclusive diagnosis, and workflow improvement.	− Quick image production. − Reduced radiation exposure. − Minimizes motion artifacts. − Automates segmentation, risk stratification, and coronary artery calcium assessment.	− Variability in diagnostic standards across institutions. − Lack of a unified gold standard. − Dependence on data sharing and standardization for widespread adoption.	− Significant initial investment is required. − Cost savings from reduced radiation use and faster imaging processes. − Financial constraints in low‐resource settings may hinder adoption.
Nuclear Cardiology	Al in PET and SPECT imaging for myocardial perfusion assessment and correlation with CT imaging.	− Enhances emission detection. − Decreases required radionuclides. − Facilitates accurate image registration − Early identification of at‐risk patients.	− Challenges similar to other imaging modalities, such as integration and standardization.	− High cost of integrating AL technology. − Potential savings from reduced radionuclide use and enhanced diagnostic accuracy. − Accessibility issues in low‐resource settings.

Abbreviations: AI, artificial intelligence; CNNs, convolutional neural networks; CT, computed tomography; DL, digital learning; ML, machine learning; PET, positron emission tomography; SPECT, single‐photon emission computed tomography.

## 4 Potential Solutions

### 4.1 Ensuring Data Privacy and Patient Confidentiality in AI‐Driven Healthcare

While AI poses a remarkable transformation to cardiac imaging, integrating AI into healthcare systems presents numerous challenges and risks related to data privacy and security [47]. One of the major challenges remains AI's reliance on access to sensitive patient data which raises significant concerns about compromising patient safety and confidentiality, especially when handling large databases [48, 49]. Addressing these concerns requires a multifaceted approach involving advanced technologies, robust security measures, and public engagement.

#### 4.1.1 Challenges and Risks

AI systems in healthcare need access to vast amounts of sensitive data to function effectively, including EHRs, imaging data, and genetic information. This extensive data usage poses several challenges:

*Data Breaches:* The risk of data breaches increases with the volume and sensitivity of data being accessed. Unauthorized access or data leaks can lead to significant privacy violations and harm patient trust [48, 49].

*Data Misuse:* Inadequate security protocols also pose the risk of misuse of sensitive health information or inadvertent exposure which creates ethical concerns.

#### 4.1.2 Technological Solutions

Several technological solutions have been proposed to enhance data privacy and security in AI‐driven healthcare:

*Blockchain Technology:* Blockchain offers a decentralized approach to data management, storing patient data across multiple nodes and reducing the risk of centralized data breaches. Although blockchain enhances data integrity and traceability, it does not eliminate the risk of data breaches [44]. It ensures that data access and modifications are recorded transparently and immutably.

*MedRec Platform:* Initiatives like the MedRec platform, developed by the Beth Israel Deaconess Medical Center and MIT Media Lab, demonstrate the feasibility of decentralized data management using blockchain technology [50]. MedRec facilitates secure access to medical records, ensuring that only authorized individuals can access patient data while maintaining an immutable audit trail of data interactions [50].

#### 4.1.3 Additional Security Measures

To protect data privacy and patient confidentiality, it is critical healthcare systems implement additional security measures alongside AI deployment [47]. These includes:

*Data Encryption:* Encrypting patient data both at rest and in transit to prevent unauthorized access. Encryption ensures that even if data is intercepted, it remains unreadable without the decryption key.

*Access Controls:* Creating strict access controls which ensure that only authorized personnel can access sensitive data. This includes but not limited to role‐based access control, where access rights are assigned based on the user's organizational role.

*Regular Audits:* Conducting regular security audits and vulnerability assessments to identify and mitigate potential security weaknesses. Audits help ensure compliance with data protection regulations and standards.

#### 4.1.4 Public Education and Advocacy

Public acceptance of data sharing for AI development is crucial. However, surveys indicate significant discomfort among adults regarding the use of their health data for AI purposes [51]. Addressing this challenge involves:

*Public Education:* Educating the public about the benefits and safeguards associated with AI in healthcare. This involves clear communication about how data will be used, stored, and protected can help alleviate concerns.

*Transparency:* Maintaining transparency about data usage and AI algorithms' functioning. Patients should be informed about how their data improve healthcare outcomes and the specific measures to protect their privacy.

*Informed Consent:* Ensuring that patients provide informed consent for the use of their data in AI development. Consent processes should be clear and understandable, emphasizing participation's voluntary nature.

By adopting advanced technologies, implementing vigorous security measures, and fostering public trust through education and transparency, we can address the data privacy and patient confidentiality challenges associated with AI in healthcare. This multifaceted approach is essential for realizing the full potential of AI in improving healthcare outcomes while safeguarding patient rights and privacy which remain primary concerns to AI integration in cardiac imaging.

### 4.2 Addressing Ethical Concerns With AI Decision‐Making

The adoption of AI in cardiac imaging and diagnosis brings forth numerous ethical dilemmas that need careful consideration to ensure patient safety, fairness, and trust in AI systems. These dilemmas revolve around privacy, security, dependability, correctness, data ownership, and biases in AI algorithms [52].

#### 4.2.1 Privacy and Security

One of the major ethical concerns is the privacy and security of patient data [42]. AI systems require extensive access to sensitive health information, raising significant concerns about data breaches and unauthorized access which could compromise patient's safety. Ensuring vigorous data protection measures is essential to maintain patient confidentiality and trust. Techniques such as data encryption, secure data storage, and strict access controls are potential solutions which may be crucial in safeguarding patient information.

#### 4.2.2 Precision and Dependability

Accuracy of AI algorithms continues to be a major concern as false diagnosis could be potentially dangerous to both patients and clinicians. Therefore, ensuring the precision and dependability of AI diagnoses is of paramount importance. Inaccurate AI‐generated diagnoses can lead to incorrect treatment plans and the potential for missed or misdiagnosed conditions, which can have serious health implications [51]. To mitigate these risks, it is essential to:

*Rigorous Validation:* Due to the consequences of potential false diagnosis with AI algorithms, it is imperative to conduct extensive validation and testing of AI algorithms using diverse and representative data sets to ensure their accuracy across different populations and clinical scenarios.

*Continuous Monitoring:* It is also important to implement systems that continuously monitor and update AI algorithms in order to adapt to new data and medical advancements, thus, ensuring they remain accurate and reliable over time.

#### 4.2.3 Interpretability of AI Algorithms

Another potential ethical challenge is the interpretability of AI algorithms. Many AI models, particularly those based on DL, operate as “black boxes,” making it difficult for healthcare providers to understand the reasoning behind their predictions [47]. This lack of transparency can hinder clinicians' ability to trust and endorse AI‐generated conclusions thus serving as a barrier to its full implementation and adoption. To address this issue:

*Explainable AI (XAI):* It is important to develop and utilize AI models that provide clear and understandable explanations for their predictions, enabling healthcare providers to interpret and validate AI decisions which can help improve trust and endorsement by the clinicians. There are currently various XAI being studied, and these are categorized into model‐specific techniques and model‐agnostic techniques. The model‐specific techniques are tailored for a specific model type and cannot be applied to other model types while the model‐agnostic technique has no specific model requirements and can be applied to a wide range of XAI models [45]. There are also several algorithms being studied such as Local Interpretable Model‐agnostic Explanations (LIME), Class Activation Mapping, Layer‐wise Relevance Propagation, and Back Propagation which fall into the post hoc interpretable techniques [45].

*Clinician Involvement:* Encourage collaboration between AI developers and clinicians which ensures AI tools are designed with practical clinical needs and interpretability in mind.

#### 4.2.4 Data Quality and Bias

Biases continue to pose significant ethical concerns in training data. If AI algorithms are trained on biased or unrepresentative data sets, they can produce skewed results that may exacerbate health disparities [53, 54]. In order to address these issues:

*Inclusive Data sets:* It is important to enhance the inclusiveness and diversity of training data sets to ensure they represent various demographic and clinical characteristics, reducing the risk of biased outcomes, especially for less represented populations.

*Bias Detection and Mitigation:* It is also important to implement techniques for detecting and mitigating biases in AI models, such as fairness constraints and bias correction algorithms, to ensure equitable treatment for all patient groups.

### 4.3 Data Ownership and Consent

Data ownership and patient consent continue to remain critical ethical considerations in AI‐integrated cardiac care. It is important patients have control over their health data and be informed on how their data will be used including in AI development. Ethical data practices include:

*Informed Consent:* Obtaining explicit informed consent from patients before their data is used for AI training and research purposes is not only a legal and ethical necessity but also a fundamental aspect of building trust in the healthcare system. With continued integration of AI into the healthcare system in the diagnosis, treatment planning, and even direct patient care, ensuring that patients fully understand how their data will be used in this context is paramount. This informed consent process must go beyond just a simple acknowledgment or checkbox; and should involve a clear explanation of the potential uses of their data, the benefits and risks associated with AI‐driven diagnostics, and the safeguards in place to protect their privacy. In addition, patients should feel empowered with the right to consent or opt‐out at any point if they feel uncomfortable with AI's involvement in their healthcare journey. The right to withdraw consent should be as easy and straightforward as giving it, ensuring that patients remain in control of their personal health information throughout their treatment. It is also critical that patients are made aware that they can change their decision without facing any penalties or repercussions regarding the quality of care they receive.

Furthermore, transparency which remains an ethical concern in AI integration in healthcare system is another critical component in this process. Patients must be provided with easily understandable information about how AI systems function, how their data will be used to train these systems, and the potential implications of AI's involvement in their care. This should be provided in easily understandable and clear terms. This transparency should also extend to the limitations and potential biases inherent in AI systems, helping patients make truly informed decisions with every possible piece of information provided. Moreover, healthcare providers and institutions should implement robust mechanisms to communicate updates about AI systems and any changes in data usage policies, ensuring ongoing patient awareness and consent.

Additionally, the consent process should be culturally sensitive and tailored to the individual needs of patients, considering factors such as language, literacy levels, and personal values. This approach not only respects the autonomy of the patient but also enhances the inclusivity of AI‐driven healthcare solutions. By involving patients in the decision‐making process, healthcare providers can foster a more patient‐centered approach to AI integration, which is essential for maintaining trust and ensuring that the benefits of AI are realized in a way that aligns with the patient's values and preferences.

*Data Governance:* It is also important to establish clear data governance policies that clearly define data ownership rights, usage permissions, and responsibilities of different stakeholders in managing patient data.

### 4.4 Addressing Ethical Challenges

A multifaceted approach is necessary to comprehensively address these ethical challenges. These include:

*Regulatory Compliance:* In order to ensure AI tools meet safety and efficacy standards, they must adhere to regulatory guidelines and best practices established by agencies such as the FDA.

*Ethical Frameworks:* It is important to develop and implement ethical frameworks that guide the development and deployment of AI in healthcare, emphasizing transparency, fairness, and accountability.

*Stakeholder Engagement:* It is critical to engage diverse stakeholders, including patients, healthcare providers, ethicists, and policymakers, in discussions about the ethical implications of AI, fostering a collaborative approach to ethical AI integration.

By proactively addressing these ethical dilemmas, we can exploit the transformative potential of AI in cardiac imaging and diagnosis while ensuring patient safety, equity, and trust in AI technologies.

### 4.5 Navigating Regulatory and Compliance Landscapes

The FDA has developed specific frameworks to ensure the safety and effectiveness of AI/ML‐based medical devices [47, 55]. The FDA's AI/ML‐based Software as a Medical Device (SaMD) Action Plan outlines the regulatory guidelines and best practices for AI/ML‐based medical devices [32]. This comprehensive plan includes key elements such as:

Provide premarket review which ensures AI/ML algorithms undergo rigorous premarket review processes to validate their safety and effectiveness.

Provide postmarket surveillance which involves the implementation of vigorous postmarking surveillance that monitors the performance of AI/ML‐based devices in real‐world clinical settings.

Provide transparency and accountability requirements for all AI algorithms in a way to ensure clinicians and regulatory bodies understand and scrutinize their decision‐making processes, to ensure more accurate results.

Create guidelines for AI systems to ensure continued learning and adaption from new data, ensuring safety in reports and effectiveness over time.

#### 4.5.1 Compliance and Collaboration

Compliance with these regulatory requirements is essential for successfully integrating AI into clinical workflows [47]. This involves:

*Collaborative Efforts:* Collaboration among clinicians, AI developers, regulatory bodies, and policymakers is critical [47]. Such collaboration ensures that AI systems are designed, tested, and implemented in ways that prioritize patient safety and clinical efficacy.

*Data Privacy and Security:* Regulatory policies and requirements that ensure compliance with the stringiest data privacy regulations such as the Health Insurance Portability and Accountability Act (HIPAA) in the USA and GDPR in Europe, are critical for the protection of patient data. It is important to implement vigorous security measures with the aim of safeguarding important and sensitive patient health information.

*Ethical Considerations:* Ethical concerns continue to limit the full integration of AI into cardiac imaging for obvious reasons. It is important to establish regulatory policies and guidelines that address biases in AI algorithms with the aim of preventing discriminatory outcomes and ensures the reports from AI algorithms are transparent, free of biases, and explainable to both patients and clinicians.

#### 4.5.2 Multifaceted Approach to AI Integration

Successfully integrating AI into cardiac imaging and diagnostic practices requires a multifaceted approach that encompasses [47]:

*Data Privacy:* It is critical to implement the stringiest data protection measures to ensure patients' confidentiality, especially with sensitive health data/information which is a major concern with AI integration with cardiac care.

*Ethical Standards:* One of the key ethical principles is equity and fairness. Measures that promote fairness, transparency, and accountability in AI‐driven decision‐making processes should be employed.

*Regulatory Compliance:* It is also critical that all regulatory requirements are met to ensure the safety effectiveness and reliability of AI for clinical use.

Overall, it is imperative to foster collaboration among all stakeholders to ensure the creation of robust security and ethical measures that safeguard patients' privacy, ensure transparency, reduce bias, promote fairness, and respect of regulatory guidelines in order to ensure the full benefits of the integration of AI into cardiac imaging. Without a doubt, the integration of AI in cardiac imaging can revolutionize the cardiology world.

## 5 Future Directions

Al's potential to reshape and significantly impact personalized cardiac imaging/medicine cannot be overemphasized. AI provides the capability for more precise risk stratification, early detection of anomaly, treatment selection, early intervention through early diagnosis thus providing key prognostic value based on individual patient characteristics. Future research should focus on developing AI models that promote the effective integration of multimodal data, including genetic, imaging, and clinical data that offers personalized insights into cardiac health of patients, disease progression, and potential outcomes.

Also, there continue to be significant challenges to full AI adoption in cardiac imaging. To enhance the full utilization and potential of AI in cardiac imaging/medicine requires a collaborative approach that requires multidisciplinary effort including the healthcare professionals, data analysts, engineers, and other pertinent stakeholders. Involving experts from different areas results in diverse contribution with the end goal of developing AI algorithms that are clinically applicable, interpretable, and resistant to errors. This also allows diverse expert interventions to tackle the current challenges that limit the full integration of AI into cardiac imaging and medicine in general.

In addition, continued advancements in research and innovation are crucial to enhance the benefits of AI in cardiac imaging while also exploring the critical areas. These include improving data quality and diversity to make the AI models more accurate and generalizable which in turn reduces bias in reports in the less represented populations. There should also be development of AI techniques that promote transparency and trust in AI‐generated diagnoses and recommendations which in turn helps promote health outcomes. Providing clinicians with real‐time feedback during cardiac imaging and diagnosis also improves decision‐making and algorithms adoption by clinicians. Future research should also focus on policies and advancements that promote seamless integration of AI algorithms with EHRs with the aim of facilitating data‐driven decision‐making and streamlined clinical workflows. Finally, addressing ethical and regulatory challenges associated with AI use in cardiac medicine such as data privacy, patient informed consent, and algorithms is critical to its full adoption to cardiac medicine.

By targeting these areas, we can harness the full potential of AI that transforms cardiac care by paving way for a new era of personalized medicine that could potentially benefit both clinicians and patients, while improving health outcomes.

## 6 Conclusion

With technological advancements, one cannot deny the potential benefits integrating AI to cardiac imaging could have on revolutionizing cardiac imaging by offering unprecedented opportunities for personalized care and improving patient outcomes. However, there are also potential barriers to its full adoption including transparency, potential bias in reports, data privacy, and ethical dilemmas. To overcome these barriers, require an interdisciplinary collaboration including patient and clinicians' education, creation of safeguards, regulatory policies, and continue research efforts. By addressing these challenges, the cardiology world can enjoy the full potential of AI in cardiac imaging and patient outcomes.

## Author Contributions

O.Z.T. contributed to the concept and design of the study. O.Z.T., F.E., O.O., and T.A. contributed to the acquisition of data. O.Z.T., F.E., and O.O. contributed to the drafting of the manuscript. O.Z.T. and T.A. contributed to revising of the manuscript. The manuscript was reviewed and approved by all authors. The requirements for authorship have been met. Each author attests to the integrity of the work.

## Conflicts of Interest

The authors declare no conflicts of interest.

## Data Availability

The authors have nothing to report.
